# Anesthetic management during adenotonsillectomy for twins with congenital insensitivity to pain with anhidrosis: two case reports

**DOI:** 10.1186/s13256-017-1406-0

**Published:** 2017-08-25

**Authors:** Cong Wang, Xianwei Zhang, Shanna Guo, Jiaoli Sun, Ningbo Li

**Affiliations:** Department of Anesthesiology, Tongji Hospital, Tongji Medical College, Huazhong University of Science and Technology, No. 1095 Jie-Fang Road, Wuhan, 430030 China

**Keywords:** Adenotonsillectomy, Autonomic nervous system dysfunction, Congenital insensitivity to pain with anhidrosis, General anesthesia, Twins

## Abstract

**Background:**

Congenital insensitivity to pain with anhidrosis is a rare autosomal recessive disorder characterized by hyperpyrexia, anhidrosis, pain insensitivity, self-inflicted injuries, and intellectual disability. The anesthetic management of these patients is challenging owing to the high risk of perioperative complications resulting from their autonomic dysfunction, such as hyperthermia, hypotension, and bradycardia, which result from autonomic nervous system dysfunction.

**Case presentation:**

Two 3-year-old Han Chinese identical male twins (weighing 13.5 kg and measuring 93 cm tall) were previously diagnosed as having congenital insensitivity to pain with anhidrosis based on clinical features and genetic screening. According to the presence of loud snoring and heavy breathing during sleep and neck radiograph findings, they were diagnosed as having tonsil and adenoid hypertrophy and needed adenotonsillectomy. Because of innate analgesia, some reports suggested that patients with congenital insensitivity to pain with anhidrosis do not require perioperative pain control. Accordingly, our patients did not receive opiates. We describe the general anesthetic management of these patients using sevoflurane and propofol, but without opiates, for adenotonsillectomy. Remarkable tachycardia and hypertension occurred during airway manipulation and when the surgical stimuli increased, and their temperatures increased from 36 °C and 36.8 °C to 37.8 °C and 38.5 °C, respectively. Patients with congenital insensitivity to pain with anhidrosis lack pain sensation, but they may have tactile hyperesthesia. Surgical noxious stimuli may therefore produce a stress response and unpleasant sensations, leading to hemodynamic fluctuation and temperature increase.

**Conclusions:**

On the basis of these findings, we suggest that careful intraoperative opiate titration may be justified to blunt the surgical stress response and promote hemodynamic and temperature stability in similar patients; we also recommend the preparation of warming and cooling devices and continuous temperature monitoring in these patients. Since anesthetic management of these patients is not simple, careful attention is required.

## Background

Congenital insensitivity to pain with anhidrosis (CIPA) is an extremely rare autosomal recessive disorder characterized by recurrent episodes of hyperpyrexia, anhidrosis, insensitivity to pain, self-mutilating behavior, and various degrees of intellectual disability [1]. CIPA is thought to result from loss-of-function mutation defects in *TRKA,* which encodes the neurotrophic tyrosine kinase receptor type 1 (NTRK1 or TRKA), a high-affinity receptor for nerve growth factor (NGF) [2]. Consequently, NGF cannot effectively bind to NTRK1, thereby resulting in reduced TRKA − NGF signaling and the death of certain NGF-dependent neurons, including nociceptive sensory and autonomic sympathetic neurons, during the embryonic stage, causing insensitivity to pain and autonomic dysfunction [3].

Consequently, patients with CIPA are susceptible to numerous anesthetic complications due to autonomic abnormalities, including hemodynamic instability, particularly hypotension, bradycardia, and hyperthermia [4]. Due to the rarity of CIPA, anesthesia management experience is limited, with previous reports describing cases of orthopedic, maxillofacial, or debridement surgery associated with disease-related trauma [5]. Here, we describe the anesthetic management of young twins with CIPA who underwent airway surgery (adenotonsillectomy) unrelated to the conditions underlying CIPA, which is normally associated with a stress response.

## Case presentation

Two 3-year-old Han Chinese identical male twins (weighing 13.5 kg and 93 cm tall) were previously diagnosed as having CIPA based on clinical features and genetic screening. The twins did not sweat or sense pain and were repeatedly admitted to the pediatric intensive care unit for hyperthermia. The older brother had signs of a cigarette burn on his chest, while the younger had a history of first metatarsal fracture without pain. Assessments performed by a trained pediatrician indicated lower-than-average language and physical development. Their clinical features are summarized in Table 1. The twins were diagnosed as having tonsil and adenoid hypertrophy based on a neck radiograph and the presence of loud snoring and heavy breathing during sleep. Consequently, their otolaryngologist suggested adenotonsillectomy. The surgery was done by the same team of experienced surgeons. Physical and laboratory examinations showed no obvious abnormalities. No sedatives were administered preoperatively.

**Table 1:** Clinical features of the patients with congenital insensitivity to pain with anhidrosis

Patients	Age at surgery (years)	Age at diagnosis (months)	Subjective symptoms	Objective findings
Male twins	3	6	1. Recurrent fever	1. Insensitive to pain
			2. Recurrent influenza	2. Delayed growth and development
			3. Chronic biting of finger nails	3. Chronic biting of finger nails
			4. Difficulty in speaking	4. Cigarette burn (older twin)
			5. Sensitivity to heat (parents’ observation)	5. Fracture (younger twin)

In the operating room, the twins’ pulse oximetry, electrocardiography, noninvasive blood pressure, and Narcotrend index (electroencephalographic measure of the depth of anesthesia) were monitored. Anesthesia was induced using 8% sevoflurane inhalation for 1 minute, and venous access was obtained immediately after they fell asleep, without signs of stress. Further anesthesia was induced with intravenously administered propofol (3 mg · kg^-1^) and cisatracurium (2 mg). Their tracheae were intubated with a cuffed endotracheal tube (internal diameter, 4.5 mm). After intubation, we monitored the invasive blood pressure, end-tidal carbon dioxide, and rectal temperature. We applied positive-pressure ventilation with a peak inspiratory pressure limit of 15 cmH_2_O and respiratory rate of 16 to 18 breaths/minute. Anesthesia was maintained with 1.5 to 2% sevoflurane and propofol infusion at 9 to 12 mg · kg^-1^ · h^-1^. Opiates were not used perioperatively. The Narcotrend index was maintained at 35 to 45. The end-tidal carbon dioxide was maintained at 35 to 45 mmHg.

**Fig. 1:**
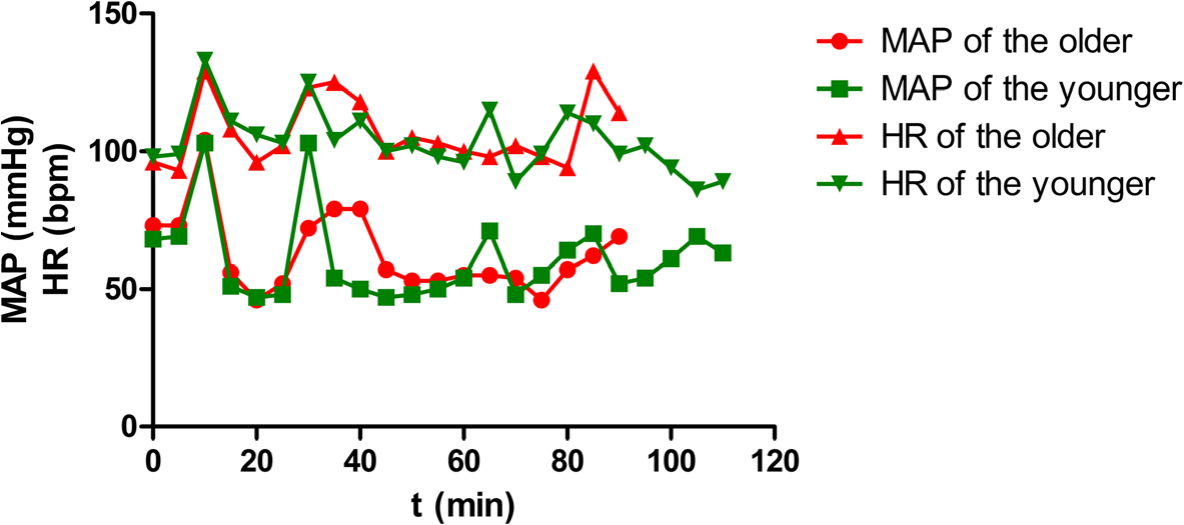
Intraoperative hemodynamic parameters of the twins. *bpm* beats per minute, *HR* heart rate, *MAP* mean arterial pressure

Hemodynamic variation was noted intraoperatively in both twins. During the intubation and surgical stimulation, their heart rate and blood pressure increased by more than 20% compared to the baseline values. Subsequently, the systolic and diastolic blood pressures were 60 to 90 and 40 to 60 mmHg, respectively, and their heart rates ranged between 80 and 100 beats per minute (bpm). Figure 1 shows our patients’ mean arterial pressures and heart rates.

Perioperatively, our patients’ temperatures increased from 36 °C and 36.8 °C to 37.8 °C and 38.5 °C, respectively. When their temperature approached 37 °C, we attempted to stabilize their body temperatures by cooling the operating room and wiping their inguinal region, popliteal fossa, and soles of their feet with alcohol. Upon leaving our post-anesthesia care unit, their axillary temperatures were 37.1 °C and 37.6 °C, respectively.

The procedures lasted 55 and 70 minutes, respectively. Follow-ups were performed 1, 6, 24, and 48 hours postoperatively. Their vital signs were stable postoperatively, and their temperatures returned to the baseline values 6 hours post-surgery. Pain was assessed with the COMFORT-Behavior (COMFORT-B) scale and Parents’ Postoperative Pain Measure (PPPM), and no significant postoperative pain was noted. They did not require opioids postoperatively.

## Discussion

Some reports have suggested that patients with CIPA do not require intraoperative analgesics because of their innate analgesia [6]. Accordingly, our patients did not receive opiates. However, remarkable tachycardia and hypertension occurred during airway manipulation and when the surgical stimuli increased, suggesting a stress response. Patients with CIPA lack pain sensation, but they may have tactile hyperesthesia [7]. Surgical noxious stimuli may therefore produce a stress response and unpleasant sensations, leading to hemodynamic fluctuation. Obviously, no pain does not mean no stress; hence, careful intraoperative opiate titration may reduce the surgical stress response and promote hemodynamic stability in these patients.

Our patients repeatedly experienced intraoperative hypotension without bradycardia, probably due to autonomic dysfunction. The norepinephrine levels in patients with CIPA are very low or even undetectable, whereas their epinephrine levels are normal [8]. In daily life, adrenal epinephrine may take over the function of norepinephrine and stabilize the blood pressure. However, anesthesia inhibits the neuroendocrine system and may reduce epinephrine secretion, thereby leading to hypotension, although the effects of propofol on blood pressure cannot be completely ruled out.

Patients with CIPA experience anhidrosis because their sweat glands lack sympathetic cholinergic innervation [9]. Hence, they are unable to regulate body temperature through sweating and radiation. Our patients’ temperatures were >37 °C despite active temperature management. Certainly, the apparent lack of hemodynamic stability and the stress response may also have contributed to their increased body temperature. Preparation of warming and cooling devices and continuous temperature monitoring are recommended for these patients.

## Conclusions

We recommend that anesthesiologists pay attention to the presence of autonomic dysfunction and perioperative complications in patients with CIPA. Using opiates intraoperatively may reduce the surgical stress response and promote hemodynamic stability in patients undergoing airway surgery. By considering the physiological characteristics of these patients, general anesthesia can be provided without major problems.

## Abbreviations

CIPA: Congenital insensitivity to pain with anhidrosis
NGF: Nerve growth factor
NTRK1: Neurotrophic tyrosine kinase receptor type 1
